# The Association between Hemoglobin A1c and the Severity of Coronary Artery Disease in Non-diabetic Patients with Acute Coronary Syndrome

**DOI:** 10.7759/cureus.6631

**Published:** 2020-01-12

**Authors:** Sultana Habib, Syed Zia Ullah, Tahir Saghir, Afaque Syed Muhammad, Zia Ud Deen, Khalid Naseeb, Rida Sherwani

**Affiliations:** 1 Cardiology, National Institute of Cardiovascular Diseases, Karachi, PAK; 2 Internal Medicine, Dow University of Health Sciences, Karachi, PAK; 3 Pediatrics, Dow Medical College, Karachi, PAK

**Keywords:** hemoglobin a1c, coronary artery disease, acute coronary syndrome, syntax score

## Abstract

Introduction

The relationship between the severity of coronary artery disease (CAD) with hemoglobin A1c (HbA1c) levels in diabetic patients is well-understood. However, the association between HbA1c and the severity of CAD in non-diabetics is still controversial. We wanted to find out if HbA1c of the non-diabetic adult population, presenting with an acute coronary syndrome (ACS), had any correlation with the severity of CAD.

Methods

We selected 119 non-diabetic adults who underwent coronary intervention for clinical reasons during the period of July 2015 to February 2017. The mean age of the patients was 54 ± 10.2 years. All patients were labeled as ‘acute coronary syndrome’, which included unstable angina, non-ST elevation myocardial infarction (NSTEMI), and ST-elevation myocardial infarction (STEMI). We obtained blood samples of patients for laboratory investigations, including HbA1c. We used the SYNTAX score as a tool to classify the severity of CAD, and patients having a SYNTAX score of >22 were considered to be having severe CAD.

Results

In order to find out the association between HbA1c and CAD, a linear regression analysis of HbA1c with the SYNTAX score was performed, which showed no statistically significant correlation between the SYNTAX score and HbA1c (correlation co-efficient = 0.142; p-value = 0.124). To compare the median value of HbA1c in groups with SYNTAX scores of ≤22 and those with SYNTAX scores of >22, we analyzed the data with the Mann-Whitney U test, which showed no significant difference in HbA1c between the two groups (p-value = 0.771). We determined the independent predictors of the severity of CAD by analyzing all variables with logistic regression, considering a SYNTAX score of >22 as a dependent variable. None of the variables, including HbA1c, proved to be statistically significant in multivariate logistic regression analysis. The unadjusted and adjusted odds ratio (OR) of HbA1c with 95% confidence intervals (CI) were 1.71 (0.47-2.92), p-value = 0.735 and 0.87 (0.33-2.29), and 0.78, respectively.

Conclusion

In conclusion, we find that HbA1c is not an independent predictor of the severity of CAD in non-diabetic adult patients.

## Introduction

Atherosclerotic cardiovascular disease (ASCVD) is a leading cause of morbidity and mortality in diabetic patients. These patients usually need strict glycemic control and continuous monitoring. Hemoglobin A1c (HbA1c) has been considered a standard criterion to monitor and diagnose diabetes mellitus (DM) for years. It has several advantages over fasting plasma glucose and oral glucose tolerance tests, including greater convenience, greater preanalytical stability, and lesser day-to-day perturbations during stress and illness [1-3]. The American Diabetes Association has recommended HbA1c as an effective diagnostic and prognostic tool for DM and its complications [3]. The complications of DM, including ASCVD, depend on many risk factors such as obesity, hyperglycemia, and dyslipidemia. The authors of Diabetes Control and Complication Trial showed the possible association between HbA1c levels and chronic diabetic complications, including cardiovascular events in type 1 DM [4]. The Atherosclerosis Risk in Communities trial showed that, among the non-diabetic adults, higher HbA1c levels lead to higher ASCVD and death [5]. A tremendous amount of work has been done to show the linear relationship between HbA1c and ASCVD in non-diabetics in past years [6-11]. Yet, few studies have shown that HbA1c is not related to the severity of coronary artery disease (CAD) in non-diabetic patients [12,13].

The association between HbA1c and the severity of CAD is still a contentious issue. In our study, we aimed to find out whether HbA1c of non-diabetic adult patients presenting with an acute coronary syndrome (ACS) had any correlation with the severity of CAD.

## Materials and methods

We conducted an observational, single-center, and cross-sectional study. We selected 119 non-diabetic adults older than 18 years who underwent coronary angiography for clinical reasons, either admitted in the emergency department or coronary care unit at the National Institute of Cardiovascular Diseases, Karachi, from July 2015 to February 2017. Our research protocol was approved by the Ethics Research Committee of our hospital and informed written consent was received from all patients. We excluded all known diabetic patients (diagnosed patients, either on or not on anti-diabetic medications, on the basis of self-reporting), patients who had increased fasting plasma glucose of ≥126 mg/dl or HbA1c of ≥6.5% (based on the American Diabetes Association diagnostic criteria) [3], those who had undergone coronary artery bypass/percutaneous coronary intervention, those who had coronary procedures other than angiography in the past, and those with hemoglobin (Hb) levels of <10.0 g/dl. Hypertension (HTN) was defined as outlined in the eighth report of the Joint National Committee on HTN, and smoking was defined according to the National Health Interview Survey [14,15]. We followed the 2013 American College of Cardiology/American Heart Association (ACC/AHA) guidelines for the treatment of blood cholesterol and the 2016 American College of Endocrinology protocol for medical care of patients with obesity to define the criteria for dyslipidemia and obesity, respectively [16,17].

All patients were labeled as ‘acute coronary syndrome’, which included unstable angina, non-ST elevation myocardial infarction (NSTEMI), and ST-elevation myocardial infarction (STEMI) according to the AHA guideline for ACS, 2014 [18]. We used the SYNTAX score as a tool to classify the severity of CAD, and patients having a SYNTAX score of >22 were considered to be having severe CAD [19,20]. We obtained a venous sample of patients for laboratory investigations, including HbA1c and fasting plasma glucose, before the procedure in the overnight fasting state for at least 8 hours. The blood samples for fasting plasma glucose were collected in fluoride vials and analyzed by the hexokinase method. An exchange chromatography method was used to detect HbA1c level, and anyone having HbA1c of ≥6.5% or fasting plasma glucose of ≥126 mg/dl was referred to the endocrinologist for further workup.

We analyzed several demographic characteristics, including age, gender, coronary risk factors, ethnicity, and body mass index (BMI). Left ventricular ejection fraction was assessed on left ventricular angiogram and echo, if available. Two experienced cardiologists reviewed the angiographic scans of the patients. We defined the CAD as significant based on the stenosis of >50% in any major vessel or its branches [21]. All significant lesions were categorized as none if no stenosis, non-obstructed (≥50-70%), or obstructed (>70-100%). Any patient having a significant disease in any of the three major coronary vessels (left anterior descending, left circumflex, and the right coronary artery) was classified as either having a single-vessel disease, 2-vessel disease, or 3-vessel disease, based on the number of vessels. Our primary endpoint was an evaluation of the relationship between baseline HbA1c level and the severity of CAD.

Statistical Analysis

Statistical package for social sciences version 19 (SPSS) (IBM, Armonk, NY) was used for data analysis. We analyzed continuous variables with the Shapiro-Wilk test to test the normal distribution of variables. All non-normally distributed variables were expressed as median with interquartile range (IQR) and analyzed with non-parametric tests. We expressed normally distributed continuous variables as mean and standard deviation, while categorical variables as frequencies and percentages. Chi-square and student t-tests were used to analyze categorical and continuous variables, respectively. A p-value of <0.05 was considered statistically significant. A Mann-Whitney U test was used to analyze the variables with a non-normal distribution. In order to find the association between the SYNTAX score and HbA1c, we analyzed the data with linear regression analysis. A univariate and multivariate logistic regression analysis of the SYNTAX score of >22 was also performed with different variables to identify the independent predictors of the severity of CAD. A p-value of <0.05 was considered statistically significant.

## Results

We had a total of 119 non-diabetic adults with a mean age of 54 ± 10.2 years, the majority of which (95, 79.8%) were males with only 24 (20.1%) females. The baseline clinical and demographic characteristics are shown in Table 1. Our demographic data also showed that most of our subjects (58, 48.7%) were overweight according to the BMI scale. The mean left ventricular ejection fraction of our patients was calculated as 50.1 ± 16.7%. 

**Table 1:** Baseline clinical and demographic characteristics of the patients ACE: angiotensin-converting enzyme; BMI: body mass index; CAD: coronary artery disease; HbA1c: hemoglobin A1c; IQR: interquartile range; NSTEMI: non-ST elevation myocardial infarction; SD: standard deviation; STEMI: ST-elevation myocardial infarction

N	119
Male, n (%)	95 (79.8%)
Age (years), mean + SD	54 ± 10.2
>50 years of age, n (%)	72 (60.5%)
BMI (kg/m^2^), mean + SD	25 ± 4.5
BMI >25, n (%)	58 (48.7%)
Waist-to-hip ratio, median (IQR)	0.99 (0.95-1.05)
Systolic blood pressure (mmHg), median (IQR)	120 (110-130)
Diastolic blood pressure (mmHg), median (IQR)	78 (70-80)
Hypertension, n (%)	50 (42%)
Dyslipidemia, n (%)	13 (10.9%)
Smoking, n (%)	53 (44.5%)
Family history of CAD, n (%)	44 (37%)
Lab investigation	
Random blood sugar (mg/dl), median (IQR)	102 (87-123)
Urea (mg/dl), median (IQR)	29 (25-37)
Serum creatinine (mg/dl), median (IQR)	1 (0.8-1.1)
HbA1c (%), median (IQR)	5.6 (5.4-6)
Medication history	
Nitrates, n (%)	84 (70.6%)
Beta-blocker, n (%)	84 (70.6%)
Statin, n (%)	94 (79%)
Calcium channel blocker, n (%)	3 (2.5%)
ACE inhibitors, n (%)	70 (58.8%)
Clinical presentation	
Unstable angina, n (%)	27 (22.7%)
NSTEMI, n (%)	62 (52.1%)
STEMI, n (%)	30 (25.2%)

We considered a SYNTAX score of >22 to indicate severe CAD and observed only 31 (26%) patients to be in that category. Most of our patients (62, 52.1%) had NSTEMI. Our data also showed that 45 (37.8%) patients had single-vessel disease, while 72 (60.5%) had either 2-vessel disease or 3-vessel disease. The coronary artery angiographic findings are shown in Table 2.

**Table 2:** Coronary angiographic characteristics of the patients CAD: coronary artery disease; IQR: interquartile range

N	119
SYNTAX score, median (IQR)	15 (8-23)
SYNTAX score >22, n (%)	31 (26.1%)
Number of vessels involved	
None, n (%)	2 (1.7%)
Single-vessel disease, n (%)	45 (37.8%)
2-vessel disease, n (%)	32 (26.9%)
3-vessel disease, n (%)	40 (33.6%)
Obstructive CAD, n (%)	91 (76.5%)
Lesion characteristics	
Discrete, n (%)	53 (44.5%)
Tubular, n (%)	57 (47.9%)
Diffuse, n (%)	35 (29.4%)
Ostial, n (%)	33 (27.7%)
Bifurcation, n (%)	16 (13.4%)
Collateral present n (%),	23 (19.3%)
Thrombus, n (%)	4 (3.4%)
Calcification, n (%)	10 (8.4%)
Tortuosity, n (%)	2 (1.7%)

**Figure 1:**
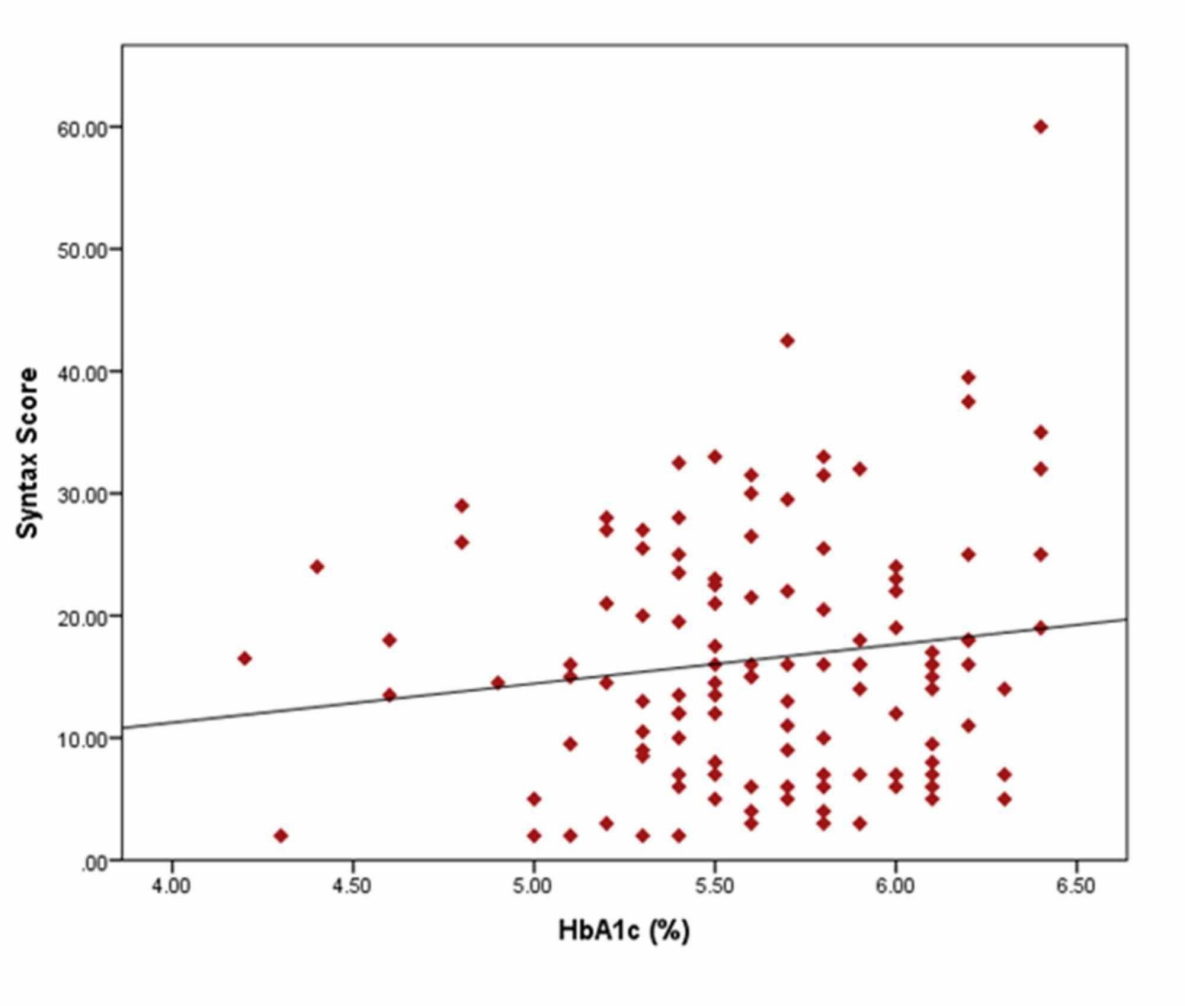
Linear regression analysis between HbA1c level and SYNTAX score in patients with ACS ACS: acute coronary syndrome

In order to find out the association between HbA1c and CAD, a linear regression analysis of HbA1c with the SYNTAX score was performed, which showed no statistically significant correlation between the SYNTAX score and HbA1c (correlation co-efficient = 0.142; p-value = 0.124). (Figure 1).

**Figure 2:**
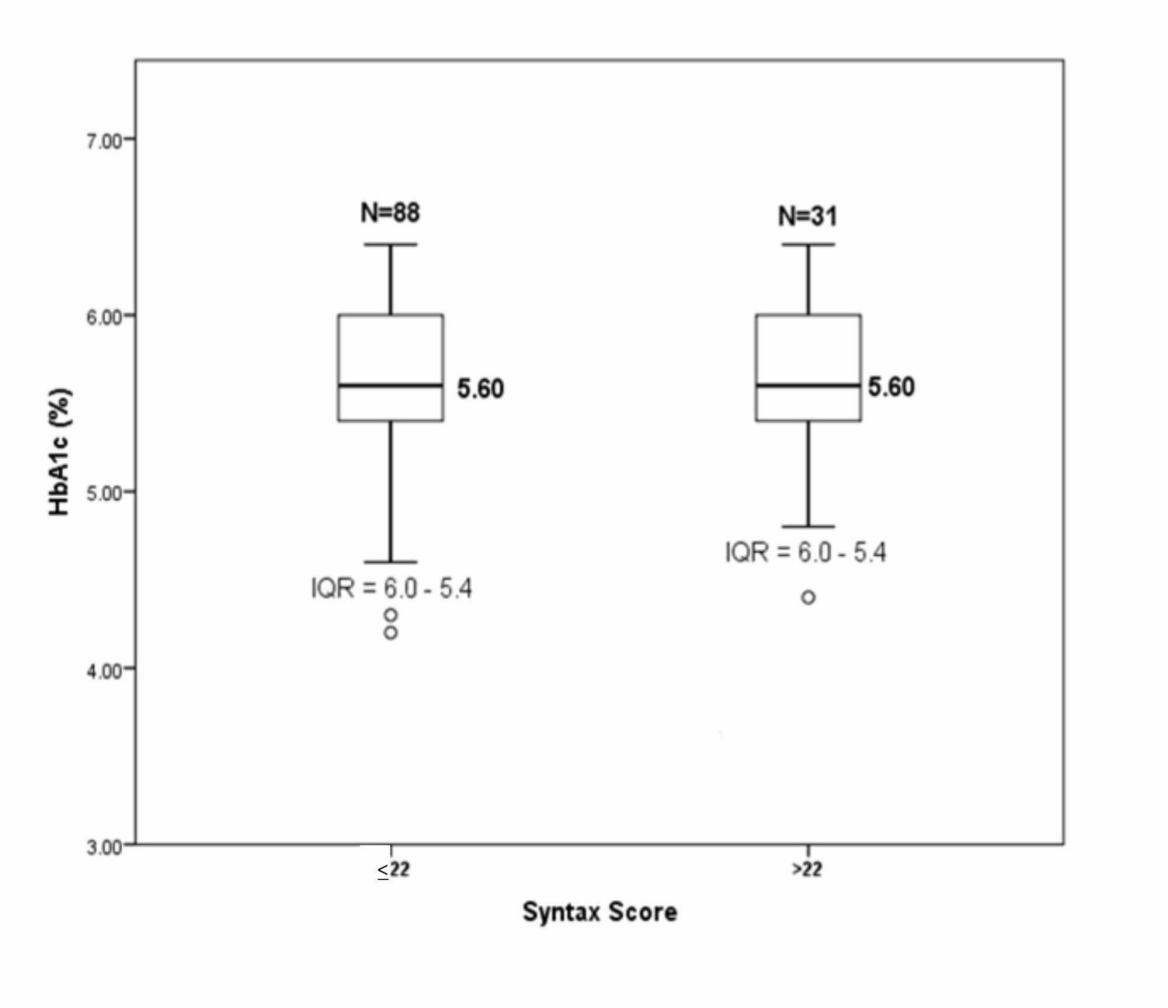
Comparison of median HbA1c in SYNTAX score groups ≤22 and >22 by Mann-Whitney U test IQR: interquartile range; HbA1c: hemoglobin A1c

To compare the median value of HbA1c in SYNTAX score groups ≤22 and >22, we analyzed the data with the Mann-Whitney U test and obtained IQR = 5.4-6.0, median = 5.6 and IQR = 5.4-6.0, Median = 5.6, respectively, with p-value = 0.771. It showed no significant difference in HbA1c between the two SYNTAX score groups. (Figure 2)

We determined the independent predictors of the severity of CAD by analyzing all variables with logistic regression, considering a SYNTAX score of >22 as a dependent variable. None of the variables, including HbA1c, proved to be statistically significant in multivariate logistic regression analysis. The unadjusted and adjusted OR of HbA1c with 95% CI were 1.71 (0.47-2.92), p-value = 0.735 and 0.87 (0.33-2.29), p-value = 0.78, respectively. (Table 3) 

**Table 3:** Predictors of the severity of CAD BMI: body mass index; CAD: coronary artery disease; CI: confidence interval; HbA1c: hemoglobin A1c; OR: Odds Ratio; STEMI: ST-elevation myocardial infarction

Predictors	Unadjusted		Adjusted	
	OR (95% CI)	P-value	OR (95% CI)	P-value
Female gender	1.22 (0.45-3.29)	0.697	0.6 (0.18-1.97)	0.397
Age	1.05 (1.01-1.09)	0.026	-	-
>50 years of age	2.86 (1.12-7.32)	0.029	2.56 (0.93-7.05)	0.069
BMI	0.97 (0.89-1.07)	0.555	-	-
BMI >25 kg/m^2^	1.17 (0.51-2.65)	0.71	1.27 (0.51-3.19)	0.606
Waist-to-hip ratio	0.02 (0-29.22)	0.303	-	-
Systolic blood pressure	1 (0.98-1.02)	0.894	-	-
Diastolic blood pressure	1 (0.97-1.04)	0.84	-	-
Hypertension	0.7 (0.31-1.61)	0.404	1.69 (0.66-4.32)	0.272
Dyslipidemia	2.07 (0.43-9.92)	0.362	0.42 (0.08-2.29)	0.317
Smoking	2.44 (1.01-5.9)	0.047	0.43 (0.16-1.18)	0.102
Family history of CAD	1.61 (0.67-3.91)	0.289	0.77 (0.28-2.1)	0.613
STEMI	0.82 (0.31-2.17)	0.695	-	-
Random blood sugar	1 (1-1.01)	0.226	-	-
Urea	0.99 (0.97-1.02)	0.723	-	-
Serum creatinine	2.26 (0.45-11.39)	0.322	-	-
HbA1c	1.71 (0.47-2.92)	0.735	0.87 (0.33-2.29)	0.78

## Discussion

HbA1c has been considered a diagnostic tool for diabetic patients for many years, and the role of HbA1c in the prediction of microvascular and macro-vascular complications of DM has been studied all over the world. A cut-off point for HbA1c to diagnose DM is ≥6.5% [3]. Yet, its relationship to the severity of CAD in non-diabetics is still unknown. Our study was designed to find out the possible association between HbA1c and the severity of CAD in non-diabetic patients with ACS. Most of the published data claimed HbA1c, even in the normal range, as a predictor of severity of CAD in non-diabetics [6-11,22-24]. However, Xinhong and some authors claimed that HbA1c was not related to the severity of CAD in either diabetic or non-diabetic patients [12,13]. Liu et al. proved that HbA1c was not a predictor of mortality in diabetic individuals, yet he showed the association of elevated HbA1c level with the mortality in non-diabetic patients with ACS [25]. A large meta-analysis conducted by Lazzeri et al. highlighted the fact that HbA1c levels were not associated with long and short-term mortality in acute STEMI patients who underwent revascularization [26]. Our results also prove that claim as wrong and stress that HbA1c is not a predictor of the severity of CAD in non-diabetic adults with ACS.

We evaluated the role of HbA1c, to assess the severity of CAD, which failed to prove any statistically significant association with the SYNTAX score. Similar to the study by Guven et al., our mean age was also low compared to other studies, which might have affected the presence of coronary risk factors and the severity of CAD in our patients [27]. The correlation analysis of our data also showed no significant association between HbA1c and the SYNTAX score. This observation clearly shows that HbA1c is not a predictor of the severity of CAD in the Pakistani adult population. The above-mentioned findings may be explained by the possibility of other confounding variables that were not considered in this observational study, such as lipid profile, sedentary life, etc. Needless to say, it warrants further evaluation by randomized trials to either confirm or disregard this observation.

Garg et al. and Ayhan et al. concluded a cut-off HbA1c level of 5.7% and 6.52%, respectively, as an independent predictor of the severity of CAD in non-diabetic patients [11, 28]. So, the best clinical threshold of HbA1c for the prediction of CAD needs to be reevaluated in non-diabetic patients with ACS. A confounding factor that affects HbA1c is the amount of Hb, as it changes the HbA1c levels [3,29]. In fact, the average Hb level of our population is either low-normal or falls in the anemic range [30]. A low-normal Hb may bring down the HbA1c level and show an association of the severity of CAD with even lower HbA1c levels. This illustration, however, has not been proved as statistically significant in our study. Nevertheless, this further stresses the need for a large trial with consideration of these parameters.

This study has many potential limitations. First, we designed a single-center study that resulted in a smaller sample size. Yet, we believe our attempt to examine the possible role of HbA1c as a predictor of the severity of CAD will likely serve as a foundation base for other researchers in Pakistan. A second limitation, which could modify the results of our study, was the low-normal average Hb of our population, which needs to be extrapolated in future researches. Another limitation was the predominance of male subjects in the study. The fourth limitation was the single measurement of HbA1c, which may not accurately reflect and may underestimate any association between HbA1c and the severity of CAD.

## Conclusions

In conclusion, we find that HbA1c is not an independent predictor of the severity of CAD in non-diabetic adult patients presenting with ACS. We also recommend large multi-center trials to further explore the questionable association between HbA1c and the severity of CAD in non-diabetic patients.
